# Presurgical Cone Beam Computed Tomography Bone Quality Evaluation for Predictable Immediate Implant Placement and Restoration in Esthetic Zone

**DOI:** 10.1155/2017/1096365

**Published:** 2017-02-22

**Authors:** Corina Marilena Cristache

**Affiliations:** Faculty of Midwifery and Medical Assisting, “Carol Davila” University of Medicine and Pharmacy, 8 Blvd Eroilor Sanitari, 050474 Bucharest, Romania

## Abstract

Despite numerous advantages over multislice computed tomography (MSCT), including a lower radiation dose to the patient, shorter acquisition times, affordable cost, and sometimes greater detail with isotropic voxels used in reconstruction, allowing precise measurements, cone beam computed tomography (CBCT) is still controversial regarding bone quality evaluation. This paper presents a brief review of the literature on accuracy and reliability of bone quality assessment with CBCT and a case report with step-by-step predictable treatment planning in esthetic zone, based on CBCT scans which enabled the clinician to evaluate, depending on bone volume and quality, whether immediate restoration with CAD-CAM manufactured temporary crown and flapless surgery may be a treatment option.

## 1. Introduction

Nowadays, cone beam computed tomography (CBCT) systems replaced multislice computed tomography (MSCT) for dental treatment and planning due to many advantages offered, including a lower radiation dose to the patient, shorter acquisition times [1, 2], affordable cost, better resolution, and sometimes greater details [3, 4]. CBCT uses isotropic voxels and, as a result, measurements are precise and considered 1 : 1; therefore study models and 3D printing or milling surgical templates can be fabricated with great accuracy [5]. Despite these preference factors, the reliability, consistency, and accuracy of CT numbers derived from CBCT imaging systems in bone quality evaluation remain controversial [6]. Therefore gray values resulting from the CBCT scan are referred to as voxel values (VVs) and not HU. The imprecision of the intensity values of CBCT systems is commonly attributed to differences in characteristics of the devices (kVp, mA, exposure time), the imaging parameters (voxel size), and the position or field of view (FOV) of the area being evaluated [7, 8].

Several studies [6–9] performed on homogenous phantoms and nonhomogenous materials (similar to human tissues) using different CBCT scanners demonstrated linear correlation between CBCT gray scale and HU.

Other studies [10–13] focused on investigating the relation between bone characteristics obtained from CBCT scan and primary stability of the implants found a direct correlation between VVs, insertion torque value (ITV), and implant stability quotient (ISQ).

Moreover, González-García and Monje [14] were the first authors to report that a strong positive correlation was present between radiological bone density (RBD) assessed by CBCT and bone density assessed by micro-CT (considered “gold-standard” for evaluating bone morphology) at the site of dental implants in the native maxillary bones. They also stated that preoperative estimation of density values by CBCT was a reliable tool to objectively determine bone density.

Based on the previous experience by González-García [14], his group also supported later the use of CBCT as preoperative tool for implant treatment planning because it was shown to be reliable to assess atrophic posterior maxilla density and microarchitecture [15].

But the final decision on the safety of immediate loading should be evaluated at the time of surgery upon measuring primary implant stability by ITV and/or ISQ.

The aim of the clinical report presented is to describe a sequence of minimally invasive treatment procedures for predictable immediate placement and restoration of a dental implant replacing a temporary maxillary canine. Decision of immediate implant placement in fresh extraction socket and restoration was based upon an CBCT evaluation of bone characteristics (volume and quality) prior to implant surgery.

## 2. Case Presentation

**Figure 1:**
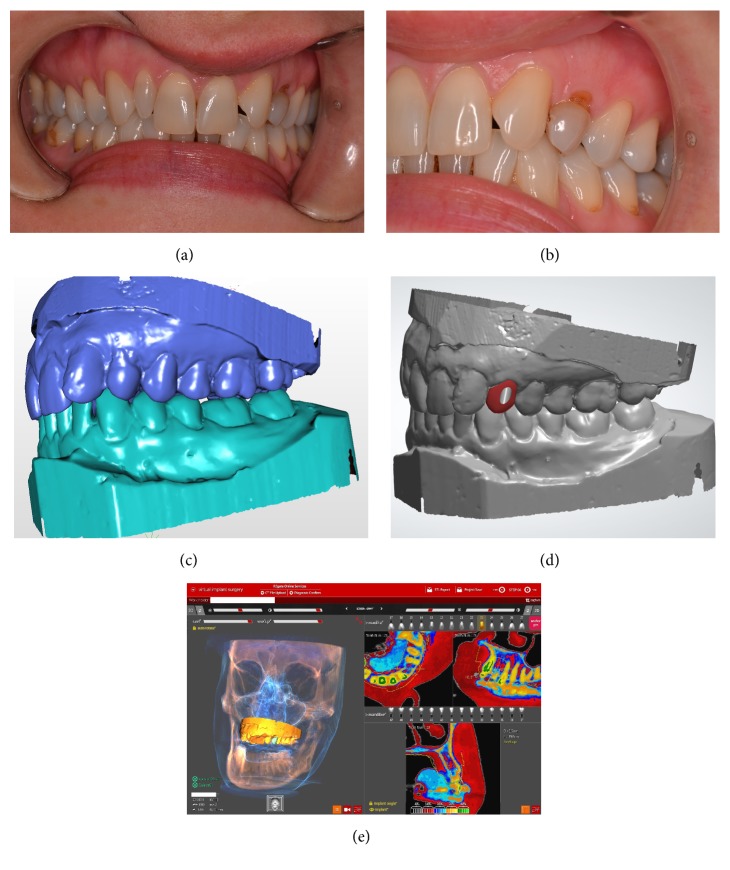
(a) At the clinical oral examination a retained #63 with complete transposition of #23 is observed. On the right maxillary arch a peg lateral incisor is present. (b) #63 with gingival margin lower than transposed #25. Gingival biotype was determined as being thick. (c) Scanned models in intercuspal position. Distovestibular rotation of transposed #23 is observed. (d) Digital wax-up of the maxillary canine implant crown according to the planned position. (e) Print-screen of the treatment plan. Bone characteristics can be observed and buccal plate can be measured in R2GATE software.

A 31-year-old woman was referred by her general dentist to our dental implant department after being evaluated by the orthodontist. The clinical and radiological examination revealed a retained upper left primary canine tooth, agenesis of #22, permanent cusped (#23) in transposition with mesiovestibular rotation. On the contralateral side a peg lateral incisor (#12) was present. Patient's request was replacing #63 with an implant and reproducing its shape with no prior orthodontic treatment. The mid-facial gingival margin of #63 was slightly lower than transposed #23 and that of the contralateral tooth (Figures 1(a) and 1(b)). The gingival tissue surrounding the crown was measured with a periodontal probe and characterized as thick [16]. Interproximal papilla was present and underlying bone level was at 1.5 mm from the margin, based on probing.

There were no medical contraindications, and patient agreed with dental implant treatment and signed the written consent form.

### 2.1. Treatment Protocol

Alginate and Tropicalgin (Zhermack, Italy) impression of the surgical site and the opposite arch for stone models was taken using standard trays. Maximum intercuspal position was registered with vinyl polysiloxane bite registration material (Regisil, Dentsply, USA). For diagnostic accuracy a radiopaque stent, R2 tray® (Megagen, Korea), was customized on the maxillary arch with nonradiopaque silicon impression material (Speedex, Coltene, Switzerland). A medium volume CBCT using ProMax 3D (Planmeca, Finland) with the characteristics of rotation of 360 degrees, height and diameter of 160 mm and 160 mm, voxel size of 0.3 mm, and the exposure factors of 110 kV and 2 mA was performed.

A series of axially sliced image data were obtained and exported to a personal computer in DICOM (Digital Imaging and Communications in Medicine) format.

Stone models alone, maximum intercuspal position, and R2 tray were scanned using a D 700 3D scanner (3Shape, Denmark) and imported as STL (Standard tessellation language) files (Figure 1(c)).

On the scan models, a virtual wax-up was designed with the use of 3Shape® CAD (Computer Aided Design) software and saved as STL file (Figure 1(d)).

### 2.2. Matching CT and Models Scan Data

DICOM files obtained from CBCT and STL files were imported in a treatment plan software R2GATE® version 1.0 (Megagen, Korea) and implant insertion was planned according to the final restoration and bone anatomy.

### 2.3. Treatment Planning

To facilitate bone quality assessment the “Digital Eye” option of R2GATE treatment planning software was used. This option provides automat conversion of CBCT gray scale in 5 basic colors, corresponding to the 256 shades of gray, from the CBCT scan, visible on computer monitors. In figures treatment plan on R2GATE software 1.0 is illustrated (Figure 1(e)).

The temporary screw-retained crown was designed according to the planned position of the implant and was sent as STL file for evaluation by the patient and the restorative team (Figure 1(e)).

### 2.4. Manufacturing of the Stereolithographic Surgical Template and Temporary Screw-Retained Crown

**Figure 2:**
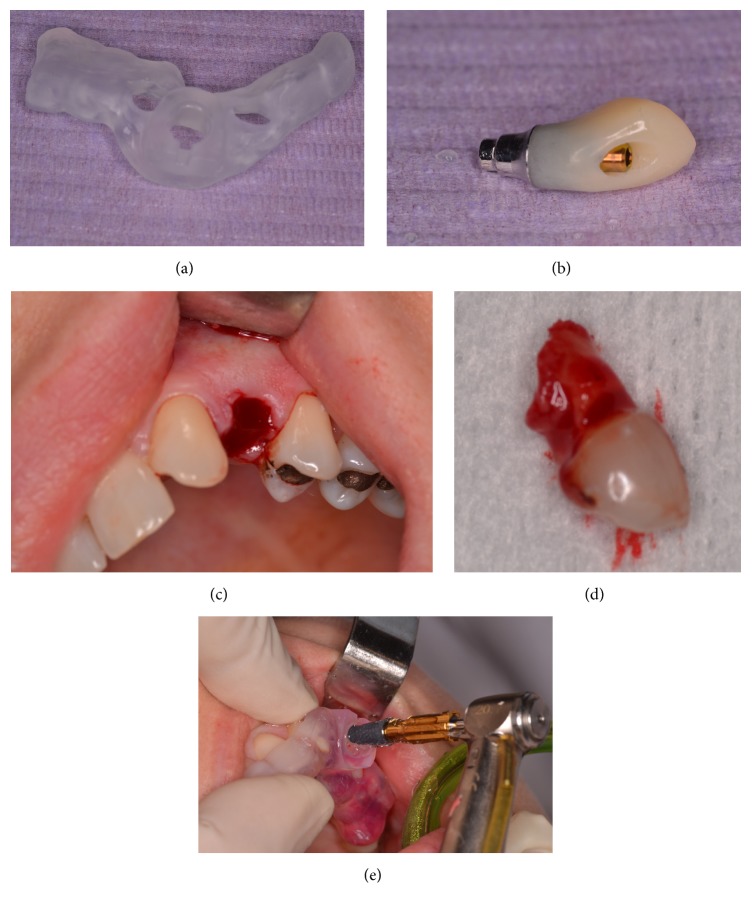
(a) 3D printed surgical template. (b) Temporary screw-retained crown manufactured before surgery according to the planned implant position. (c) Atraumatic extraction of #63. (d) #63 after extraction with almost no root resorption. (e) Flapless dental implant insertion utilizing the stereolithographic surgical template.

Surgical template was printed according to the established position of the implant, which was simulated in alveolar bone by the R2GATE software based on the obtained CBCT data, estimated bone quantity and quality, and digital wax-up of the future prosthetic reconstruction. Screw-retained provisional was manufactured according to the planed implant position and delivered before surgery with the computer aided design and manufacturing (CAD-CAM) surgical template (Figures 2(a) and 2(b)).

### 2.5. Surgery and Provisional Crown

Atraumatic extraction of the primary canine using periotomes was performed (Figures 2(c) and 2(d)). Care was taken not to damage the labial bone, the socket was irrigated with saline, and the site was examined to verify an intact buccal plate. A 10 mm with 3,5 mm diameter Megagen AnyRidge® (Korea) was inserted flapless, under local anesthesia, according to the planned 3D position with the use of the stereolithographic template (Figure 2(e)).

**Figure 3:**
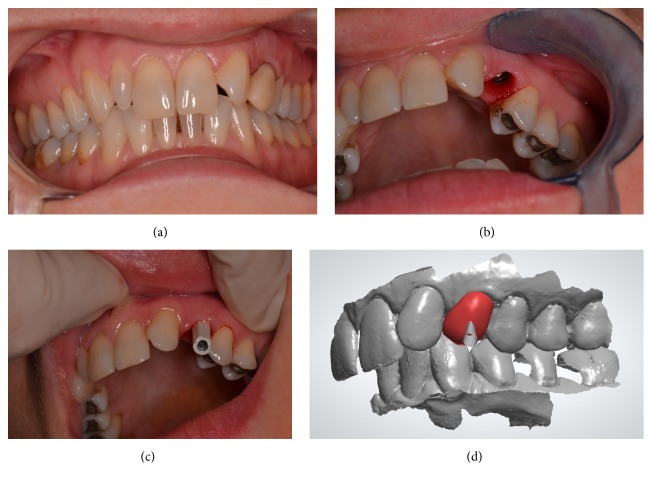
(a) Patient after surgery with the temporary crown screwed into the implant. (b) Eight weeks after implant surgery, temporary crown was removed and dentogingival complex was successfully preserved. (c) Digital impression with scannable coping screwed into the implant. (d) Digital wax-up of the final crown.

Insertion drill sequence was recommended by the manufacturer according to the bone characteristics evaluated with the aid of the CBCT in order to acquire maximum bone to implant contact. Torque insertion value was 65N cm resulting in a good primary stability. The space between the inner surface of the labial osseous wall and the labial surface of the implant was filled with resorbable bovine bone graft material (Cerabone, Botiss, Germany). After implant insertion the prefabricated provisional crown was screwed into the implant and occlusal adjustments were performed (Figure 3(a)).

Eight weeks after implant surgery, after uneventful osseointegration, the provisional crown was unscrewed and an excellent healing of dentogingival complex and papilla preservation were observed (Figure 3(b)). Digital impression was performed (Figure 3(c)) and a CAD-CAM zirconia customized abutment and ceramic crown were manufactured according to patient's request (Figure 3(d)).

**Figure 4:**
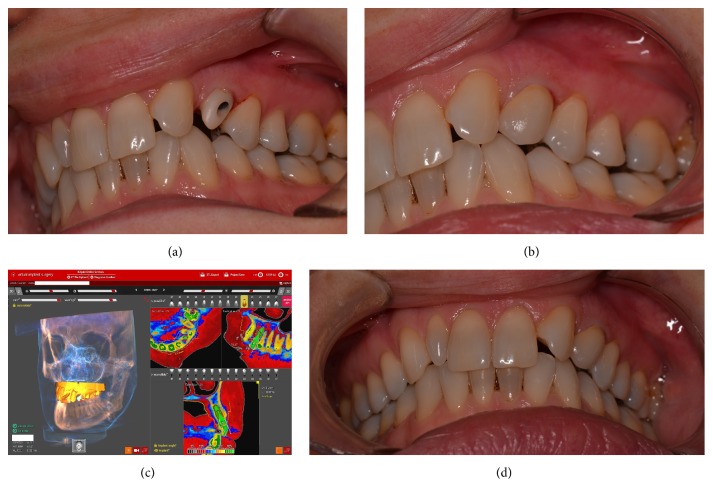
(a) Zirconia customized abutment screwed on the implant. (b) Final full ceramic crown cemented. (c) CBCT at one-year follow-up. No bone resorption was noticed. (d) Final crown at 1-year follow-up. Distal and mesial papilla are present. PES and WES scored 14/14 and 9/10, respectively. WES score is 9 due to nonperfect resemblance of the restored canine. The patient's option was reproducing a canine crown resembling a premolar and not #13.

Patient was very pleased with the final result (Figures 4(a) and 4(b)).

At the annual recall the implant showed no signs of complications nor infection (Figure 4(c)). Clinical assessment of pink esthetic score (PES) [17] and white esthetic score (WES) [18], utilized to objectively evaluate single tooth implant restoration, rated 14/14 and 9/10, respectively, due to minor discrepancies between the two canines (left and right). On the CBCT evaluation no buccal bone resorption was observed after one year of function (Figure 4(d)).

## 3. Discussions

Primary implant stability is the key factor for immediate restoration and it is obvious that attention should be paid to the local bone quantity and quality during the presurgical planning phase [19].

For bone volume, it is well known that CBCT provides submillimeter isotropic voxels allowing accurate measurements, with minimal magnification and distortion (error less than 0.1 mm) [4], allowing safe dental implant insertion [20, 21].

Bone quality on CBCT, prior to implant placement, even though not being quantifiable in reproducible unit (e.g., HU), can be reliably evaluated by assessing radiographic bone density (RBD) as demonstrated by González-García and his group in both nonatrophic [14] and atrophic maxilla [15]. The authors compared architectural metric parameters, bone volume (BV) and total volume (TV) on micro-CT bone biopsies at implant sites to radiologic bone density (RBD) measured on CBCT, and found a high positive correlation between RBD and BV/TV (*r* = 0.858) [14]. Moreover, they established, in pristine maxillary bone, some regression equations allowing clinicians to preoperatively estimate the microstructure of the maxillary bone based on a mean bone density value assessed by CBCT [22].

According to González-García and Monje [14], preoperative estimation of density values by CBCT is a reliable tool to objectively determine bone density. Therefore a temporary crown can be manufactured prior to implant insertion to facilitate immediate implant restoration especially for the high requirements in esthetic zone [23].

The implant treatment planning software utilized (R2GATE) allowed the clinician to better evaluate bone quality by using “Digital Eye” option. Due to the fact that the eye and the monitor display are not able to handle 4096 (2^12^) shades of gray obtained from a CBCT scan (computer monitor is able to display only 255 shades of gray and human eye can clearly distinguish between 8 and 16 [24]), the R2GATE software automatically converts gray shades, measuring X-ray absorption, in a range of 5 basic colors. Human eye sensitivity is limited for gray shades but is able to distinguish 128 fully saturate hues and with the addition of white light to hue enables discernment of a number of 350.000 shades, 20.000 times more than shades of gray [24, 25].

Intensity transformations are the most commonly used image processing techniques, enabling image data adjustments for better visualization. Therefore a scale has to be mapped to display intensity values that extends from 0 to 255 and the conversion is usually done with a linear function. For example, a function to convert the voxel values lying between a lower limit *A* and an upper limit *B* to a scale of 255 gray values has a window with *W* = *B* − *A* and a window level (or center) *L* = (*A* + *B*)/2. As the window with *W* decreases, the contrast in the displayed image increases. As the window level (*L*) moves up (down), the image becomes darker (lighter). This operation is called windowing (leveling) and adjusts brightness and contrast for a better visualization, without changing the original data of the CBCT [26]. Windowing allows a better evaluation of voxel values (VVs) from the CBCT, facilitating predictable treatment planning. R2GATE software, used for treatment planning in the presented case report, allows changing the values of colors displayed on the screen (contrast control) using windowing in order to better visualize the volume of interest, outbalancing the limitations of human eyes and computer monitors.

Moreover, the color-coded bone density assessment enables the clinician to establish an individualized drilling protocol in order to improve dental implant primer stability.

The use of a guided surgical approach through a computerized simulation enables the implant placement to be provided with around 98% accuracy [27, 28]. Guided surgery is advantageous for conventional implant placement, immediate implant placement, and potential immediate provisionalisation.

The advantage of single stage immediate implant placement is more predictable preservation of the peri-implant gingival tissue [29] with less patient discomfort and less treatment time [30].

The criteria and techniques for proper immediate implant placement have previously been established and reported with successful long-term outcomes [31, 32]. Some aspects for treatment's success are mandatory: at least 2 mm of buccal plate to avoid soft and hard tissue recession [33], positioning the implant with sufficient primary stability in the extraction socket [32], without flap elevation, thick gingival biotype if possible [34, 35], ideally 3D positioning of the implant, grafting the gap between the buccal wall and the implant [36], and using a provisional crown immediately after implant insertion for maintaining soft tissue contours [31].

In order to compensate for the expected horizontal bone resorption of the buccal plate, the use of bone substitutes, with a low resorption rate, to fill the gap has been shown to reduce this resorption significantly and therefore their use should be advocated when the esthetic demands are high [37].

Immediate implant placement and restoration not only reduced the number of necessary surgeries but also decreased treatment time and costs and is recommended to be utilized each time local and systemic condition permits [38].

## 4. Conclusions

This case report presented step by step a straightforward, predictable, treatment planning, based on CBCT scans, which enables the clinician to evaluate whether immediate restoration and flapless surgery may be a treatment option and allows CAD-CAM manufacturing of a temporary crown with adequate subgingival contour in order to preserve soft tissue architecture.

The decision of immediate implant placement and manufacturing provisional crown can rely on CBCT bone quality assessment during the presurgical implant-planning phase [19].

The use of CBCT gray scale automate conversion in 5 colors and the windowing process allows the clinician for a better evaluation of bone characteristics for a precise implant planning and crown fabrication. But final decision on immediate restoration can be taken only at the time of surgery, after objective evaluation of primary implant stability.
